# Involvement of multiple phytoene synthase genes in tissue- and cultivar-specific accumulation of carotenoids in loquat

**DOI:** 10.1093/jxb/eru257

**Published:** 2014-06-16

**Authors:** Xiumin Fu, Chao Feng, Chunyan Wang, Xueren Yin, Pengjun Lu, Don Grierson, Changjie Xu, Kunsong Chen

**Affiliations:** ^1^Laboratory of Fruit Quality Biology/The State Agriculture Ministry Laboratory of Horticultural Plant Growth, Development and Quality Improvement, Zhejiang University, Zijingang Campus, Hangzhou 310058, China; ^2^South China Botanical Garden, Chinese Academy of Sciences, Xingke Road 723, Tianhe District, Guangzhou 510650, China; ^3^Plant & Crop Sciences Division, School of Biosciences, University of Nottingham, Sutton Bonington Campus, Loughborough LE12 5RD, UK

**Keywords:** Carotenoid, function, loquat (*Eriobotrya japonica*), mutation, phytoene synthase, tissue-specific expression.

## Abstract

Differences in carotenoid accumulation between tissues and cultivars is common in plants. White-fleshed loquat cultivars had low levels of carotenoids in the flesh, but accumulated carotenoids in peel when ripe, and the leaves accumulated similar carotenoids to those in the red-fleshed loquat cultivars. The catalytic activity and expression patterns of four phytoene synthase (*PSY*) genes, *EjPSY1*, *EjPSY2A*, *EjPSY2B*, and *EjPSY3*, were analysed to understand their roles in different loquat (*Eriobotrya japonica* Lindl.) types. *EjPSY1* was responsible for carotenoid synthesis in the fruit peel but not the flesh, whereas *EjPSY2A* was responsible for carotenoid accumulation in flesh of ripening fruit. A mutant *EjPSY2A* ^*d*^, with the same tissue specificity and expression level as *EjPSY2A*, but lacking the C-terminal region and corresponding catalytic activity, was discovered in white-fleshed varieties, explaining the lack of carotenoids in the white flesh. The catalytic role of *EjPSY2B* was most significant in leaves. The tissue-specific expression of *EjPSY1* and *EjPSY2B* explained well how peel and leaf tissues can still accumulate carotenoids in white-fleshed cultivars, which have lost the functional EjPSY2A. *EjPSY3* mRNA abundance was ~1000-fold less than that of other *PSY* mRNAs in all tissues examined. In addition, neither the normal sized transcript nor two alternatively spliced forms, *EjPSY3α* in LYQ and *EjPSY3β* in BS cultivars, encoded functional enzymes, and it is concluded that *EjPSY3* plays no role in carotenoid accumulation. In addition, it was noted that recruitment of *PSY* genes for expression in specific tissues of different plants has occurred independently of gene structure and evolutionary origin.

## Summary

Four phytoene synthase genes and several variants were characterized, and their evolution and function in differential carotenoid accumulation in leaf, peel, and flesh of white- and red-fleshed loquats were established.

## Introduction

In addition to being important accessory pigments in photosynthesis (Demmig-Adams and Adams, 1992, 2002; Niyogi, 1999), carotenoids also form the basis of many flower and fruit colours that attract animals and facilitate pollination and seed dispersal (Howitt and Pogson, 2006). Dietary carotenoids are important for human health, some as essential precursors to vitamin A and others as antioxidants and anticarcinogenic agents, as well as having cardiovascular and eye disease-preventing bioactives (Krinsky *et al.*, 2003; Clagett-Dame and Knutson, 2011; Abdel-Aal *et al.*, 2013).

Carotenoids are synthesized by all photosynthetic organisms, mainly plants, and some non-photosynthetic bacteria, as well as certain fungi and a few species of animals (Moran and Jarvik, 2010). In view of their important roles, carotenoid biosynthesis in higher plants has been widely studied and the pathway has been elucidated in the past two decades (Hirschberg, 2001; Fraser and Bramley, 2004; DellaPenna and Pogson, 2006; Chen and Wurtzel, 2010; Ma *et al.*, 2013; Shumskaya and Wurtzel, 2013).

Diverse mechanisms have been proposed to be responsible for the different carotenoid patterns among cultivars. In some cases, these can be explained by sequence mutation of the gene participating in the carotenoid biosynthetic pathway, either inside the open reading frames (ORFs), resulting in an increase or decrease, or even a loss of activity of the encoding enzyme, or in the promoter region, resulting in changes in the level of expression. For example, a null mutation in the B gene in *old-gold* (*og*) tomato causes the fruit to accumulate a higher ratio of lycopene to β-carotene, while mutations in the promoter of the B gene increased the level of expression and resulted in a lower ratio of lycopene to β-carotene in *Beta* tomato (Ronen *et al.*, 2000). Similarly, four naturally occurring *lcyE* polymorphisms explained the variation in α-carotene and β-carotene biosynthetic branches and the resulting differences in provitamin A compounds in maize (Harjes *et al.*, 2008); the carotenoid cleavage dioxygenase 4 (*PpCCD4*) alleles of yellow peach and white peach have arisen from different ancestral haplotypes by at least three independent mutational events (Falchi *et al.*, 2013). Progress in plastid biology has provided other insights into mechanisms affecting carotenoid content. Carotenoid accumulation has been shown in some cases to be related to plastid biogenesis and interconversion (Egea *et al.*, 2010), as in the high β-carotene content in the orange curd of a cauliflower mutant, which was shown to be due to the differentiation of proplastids and other non-coloured plastids into chromoplasts caused by the *Or* gene (Paolillo *et al.*, 2004; Lu *et al.*, 2006). Similarly, Kilcrease *et al.* (2013) demonstrated a linkage between chromoplast architecture and carotenoid composition in diverse *Capsicum annuum* fruit by using multiple microscopic approaches.

A sequence mutation or alteration in the mRNA level of phytoene synthase (*PSY*), the first committed step in the carotenoid pathway, could strongly affect plant carotenoid accumulation. Truncation of PSY1 was shown to lead to lack of carotenoid accumulation in tomato fruit (Fray and Grierson, 1993), and overexpression of *PSY1* in tomato resulted in changes in pigmentation and plastid type (Fraser *et al.*, 2007). In addition, numerous reports showed that *PSY* can have several family members, each, to some extent, with its own tissue-specific expression. As an example, the second phytoene synthase, *PSY2*, in tomato was shown to be predominantly responsible for carotenoid formation in chloroplast-containing tissues (Fraser *et al.*, 1999). Recently, a third member of the *PSY* family, *PSY3*, was discovered from genome sequencing, namely *SlPSY3* (Kachanovsky *et al.*, 2012) and *CitPSY3* (Peng *et al.*, 2013). These third members showed fewer transcripts among all the tissues studied and their roles in carotenoid biosynthesis are largely unknown. In general, there has not been a great deal of research on the different *PSY* family member genes in dicot plants, probably because the model plant *Arabidopsis* only has one *PSY* gene.

Some fruits of dicot plants, such as loquat (Zhou *et al.*, 2007; Fu *et al.*, 2012), can accumulate carotenoids in both peel and flesh, but with considerable variation between cultivars. The white-fleshed loquat cultivars have few carotenoids in the flesh and appear ivory, but the peel is a yellow colour when ripe. In a previous study (Fu *et al.*, 2012), it was discovered that the differential expression of carotenogenic genes was insufficient to explain the large difference in carotenoid content between the red- and white-fleshed cultivars, indicating that there may be another regulatory mechanism underlying this phenomenon. In the present study, a truncated and non-functional mutant, *EjPSY2A* ^*d*^, was identified in the genome of white-fleshed loquat cultivars and shown to explain the low levels of carotenoids accumulated in the flesh of white-fleshed loquat. The tissue-specific expression patterns of *EjPSY1* and *EjPSY2B* explained well how peel and leaf tissues can still accumulate carotenoids in white-fleshed cultivars, which have lost the functional *EjPSY2A* expressed in fruit flesh. It was speculated that *EjPSY3* is functionless in loquat. In addition, it was observed that *PSY* expression patterns in dicot plants are independent of gene structure and evolution.

## Materials and methods

### Plant materials

Luoyangqing (LYQ, red-fleshed) and Baisha (BS, white-fleshed) loquats (*Eriobotrya japonica* Lindl.) were sampled from an orchard in Luqiao, Zhejiang, China. For analysis of different plant tissues and fruit at different developmental stages, young leaves, mature leaves, roots, stems, and petals at anthesis were collected from each cultivar. Fruit of six developmental stages, S1, fruitlet, 45 days after full bloom (DAFB); S2, immature green, 75 DAFB; S3, mature green, 95 DAFB; S4, breaker, 105 DAFB; S5, half ripe, 110 DAFB; and S6, ripe, 115 DAFB, were collected. Mature fruits and leaves of other red-fleshed (Jiajiao, Baozhu, Dameiguihongpao, Dayeyangdun, and Dawuxing) and white-fleshed (Tianzhong, Bingtangzhong, Ninghaibai, Guanyu, Baiyu, and Biqi) cultivars were picked from the Fruit Technology Extension and Service Center in Taihu, Jiangsu, China. All tissue samples were immediately frozen in liquid nitrogen and stored at –80 °C until further use. Each sample, except for that for genomic DNA extraction, consisted of three biological replicates.

### Carotenoid extraction, quantiﬁcation, and HPLC analysis

Carotenoids were extracted from tissues and analysed by high-performance liquid chromatography (HPLC), according to a method previously described by Xu *et al.* (2006).

### Searching for and cloning the *PSY* gene family members in loquat

RNA-Seq libraries of LYQ and BS fruits were constructed (unpublished data). Nine *PSY* unigenes in the LYQ library and two *PSY* unigenes in the BS library (Supplementary Fig. S1 available at *JXB* online) were obtained by either searching the libraries with the name of the gene or BLASTing with the homologous sequences from the model plants *Arabidopsis*, rice, or tomato. Further sequence alignment analysis was carried out by the BLAST program online (http://www.ncbi.nlm.nih.gov/BLAST) and Clustal X (1.81), and these 11 unigene sequences were assembled into three *PSY* unigenes in LYQ and one *PSY* unigene in BS. The genomic database of Rosaceae species (http://www.phytozome.net/) of apple, strawberry, and peach was also used to search the *PSY* sequences, and degenerate primers (forward primer, 5′-CTTCCAAATGTGTTCTACAATTTC-3′; reverse primer, 5′-TGTTTTTATTATTGGGACATCAA-3′) were designed based on sequences corresponding to a highly conserved peptide in order to clone *EjPSY3* from loquat.

### Isolation of RNA

Total RNA was extracted from frozen powder following a previously published protocol (Fu *et al.*, 2012). RNA integrity was electrophoretically verified with ethidium bromide staining and purity by checking that the *A*_260_/*A*_280_ absorption ratio was between 1.9 and 2.1. Potential contamination with DNA was eliminated by treatment with DNase I (RNase-free) (Fermentas MBI).

### Full-length cDNA amplification and sequence analysis

Three *PSY* unigenes in LYQ and one *PSY* unigene in BS were amplified to obtain the full-length sequences using 5′RACE (rapid amplification of cDNA ends) and 3′RACE primers (Supplementary Table S1 at *JXB* online) using the SMART™ RACE cDNA amplification Kit (Clontech). Three *EjPSY* gene family members (*EjPSY1*, *EjPSY2A*, and *EjPSY2B*) were obtained from the LYQ library and one mutant *EjPSY2A* ^*d*^ was obtained from the BS library. The forward and reverse primers 5′-CTTCCAAATGTGTTCTACAATTTC-3′ and 5′-TGTTTTTATTATTGGGACATCAA-3′ were used to obtain the full-length *EjPSY3* sequence. The cDNAs of the *PSY* genes were aligned and a phylogenetic tree constructed using MEGA (version 5.0), and the sequence alignment was used for a Neighbor–Joining (NJ) tree using default parameters in MEGA. Bootstrap analysis of the NJ tree was performed using 1000 replicates.

### DNA extraction and *EjPSY* genomic sequence amplification

Genomic DNA was extracted from young leaves by the CTAB (cetyltrimethylammonium bromide) method as described by Huang *et al.* (2013). The genomic DNA sequences of *EjPSY1*, *EjPSY2A*, and *EjPSY2B* were cloned by genomic walking using gene-specific primers (Supplementary Table S2 at *JXB* online). The complete genomic sequences were obtained using primers (Supplementary Table S3 at *JXB* online). Another specific primer pair was designed to identify the genomic DNA sequence (Supplementary Table S4 at *JXB* online) of *EjPSY2A* and *EjPSY2A* ^*d*^.

### Real-time reverse transcription–PCR (RT–PCR)

A 3 μg aliquot of total RNA was reverse transcribed using the M-MuLV Reverse Transcriptase kit (Fermentas MBI) and oligo(dT) primers according to the manufacturer’s instructions. Gene-specific primers for *EjPSY1*, *EjPSY2A*, *EjPSY2A* ^*d*^, *EjPSY2B*, *EjPSY3*, *EjPSY3α* and *EjPSY3β* were designed and tested for specificity (Supplementary Table S5 at *JXB* online). The real-time quantitative PCR was conducted in a Roche LightCycler thermocycling real-time PCR system. The expression level of actin was used to normalize the mRNA levels for each sample, with abundance expressed as a multiple of actin (Fu *et al.*, 2012).

### Plasmids and functional complementation

The pAC-85b plasmid contains all genes required for β-carotene production except for *PSY* and can be used for testing *PSY* activity (Cunningham and Gantt, 2007). The pAtPSY plasmid containing the *PSY* of *Arabidopsis* was used as a positive control in the experiment. When transformed together with pAC-85b and pAtPSY, *Escherichia coli* DH5α cells could accumulate the end-product β-carotene. The empty vector was constructed by a deletion of the *Eco*RΙ*–Eco*RΙ *AtPSY* fragment from this plasmid. pAC-85b was used for heterologous complementation to test the function of *EjPSY1*, *EjPSY2A*, *EjPSY2A* ^*d*^, *EjPSY2B*, *EjPSY3*, *EjPSY3α*, and *EjPSY3β.* cDNAs were subcloned as in-frame translational fusions as follows: *EjPSY1*, *EjPSY2A*, *EjPSY2A* ^*d*^, and *EjPSY2B* were amplified using primers (Supplementary Table S6 at *JXB* online) with a *Sal*Ι site (bold) in the forward primers and an *Eco*RΙ site (bold) in the reverse primers; *EjPSY3*, *EjPSY3α*, and *EjPSY3β* were amplified using primers (Supplementary Table S6 at *JXB* online) with a *Bam*HΙ site (bold) in the forward primer and a *Sal*Ι site (bold) in the reverse primer. The above amplified cDNAs were subcloned into the corresponding sites of the digested pAtPSY plasmid, and renamed pEjPSY1, pEjPSY2A, pEjPSY2A^d^, pEjPSY2B, pEjPSY3, pEjPSY3α, and pEjPSY3β, respectively.

Chemically competent pAC-85b-containing *E. coli* DH5α cells were prepared and transformed with pEjPSY1, pEjPSY2A, pEjPSY2A^d^, pEjPSY2B, pEjPSY3, pEjPSY3α, and pEjPSY3β. A single colony was used to inoculate a 2ml Luria broth (LB) culture, supplemented with ampicillin (100 μg g^–1^) and chloramphenicol (50 μg g^–1^), grown at 37 °C and 180rpm. The overnight culture was then used to inoculate a 50ml LB culture containing the same antibiotics, which was maintained at 37 °C with shaking for 1 d, and cells were harvested by centrifugation at 5000 *g* for 15min. After photographing, total carotenoids were extracted from the bacterial cell pellets and analysed by HPLC by the method described above.

## Results

### The carotenoid accumulation patterns in the tissues of different red-fleshed and white-fleshed cultivars are grouped into two types

**Fig. 1.**
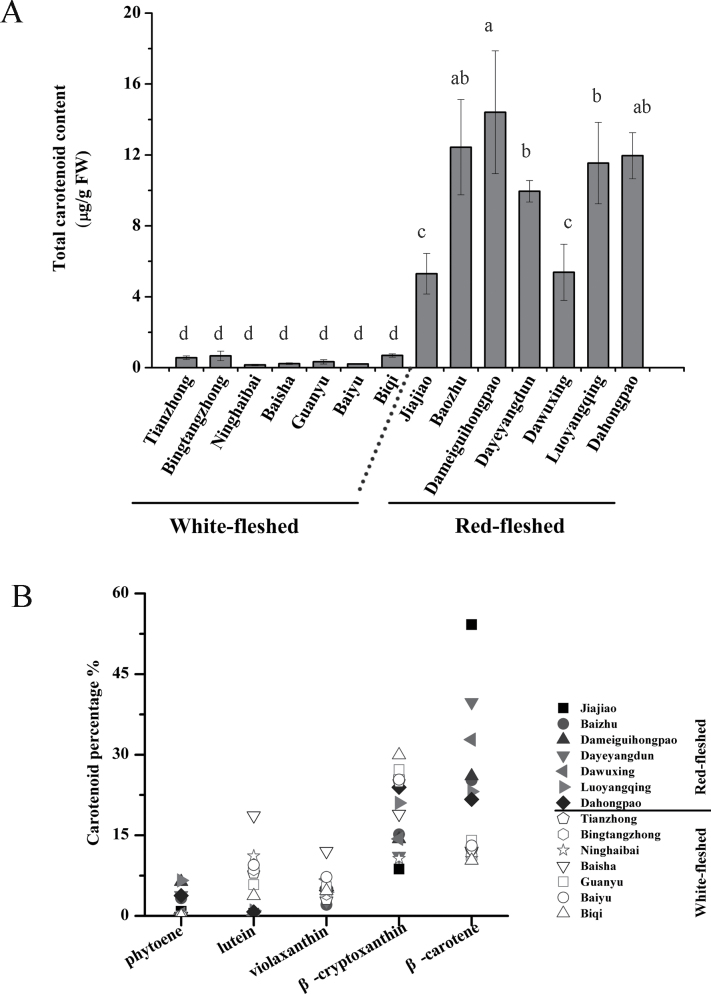
Analysis of the carotenoid content and composition in flesh of different red-fleshed and white-fleshed loquat cultivars. (A) Total carotenoids were extracted from the flesh of red-fleshed [Jiajiao, Baozhu, Dameiguihongpao, Dayeyangdun, Dawuxing, Luoyangqing (LYQ), and Dahongpao] and white-fleshed [Tianzhong, Bingtangzhong, Ninghaibai, Baisha (BS), Guanyu, and Biqi] cultivars and quantified by HPLC. Significance was set at a *P*-value <0.05. (B) The percentage composition of the main carotenoids (phytoene, lutein, violaxanthin, β-cryptoxanthin, and β-carotene) in the flesh of red- and white-fleshed loquat cultivars; the absolute amount are provided in Supplementary Table S7 at *JXB* online.

**Fig. 2.**
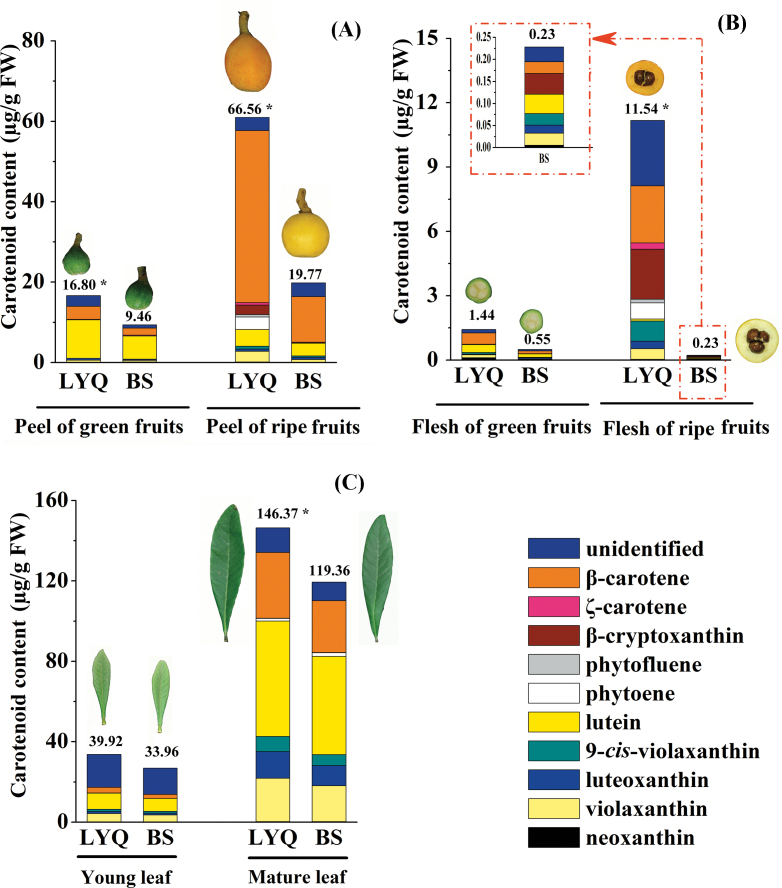
Carotenoid analysis in the tissues of LYQ and BS. (A) Carotenoid content and composition were compared in the peel of Luoyangqing (LYQ) and Baisha (BS) at the green and ripe stages. (B) Carotenoid content and composition were compared in the flesh of LYQ and BS at green and ripe stages. (C) Carotenoid content and composition were compared in young and mature leaves of LYQ and BS, respectively. The total carotenoid content is shown at the top of the column, with an asterisk indicating a significant difference, at a *P*-value <0.05, between the two cultivars. The absolute amount are provided in Supplementary Table S8 at *JXB* online.

The flesh of red-fleshed cultivars appeared red-orange due to the accumulation of abundant carotenoids [5.30–14.41 μg g^–1^ fresh weight (FW)]. In contrast, far fewer carotenoids (0.16–0.69 μg g^–1^ FW) accumulated in the flesh of white-fleshed cultivars (Fig. 1A). Regarding individual carotenoids in flesh, phytoene was present only in red-fleshed cultivars, and the amounts of violaxanthin, β-cryptoxanthin, and β-carotene were much lower in white-fleshed cultivars (Supplementary Table S7 at *JXB* online). Although no significant difference in the amount of lutein in the flesh was observed between the two groups (Supplementary Table S7 at *JXB* online), the percentage of lutein in the total carotenoids in the flesh was much higher in white-fleshed cultivars (averaging 9.98%) than in red-fleshed cultivars (averaging 0.33%) (Figs 1B, 2B). In peel, the total amount of carotenoids in green LYQ was ~1.5-fold higher than in BS, but the carotenoid profiles were almost the same, and comprised mainly lutein (60% on average) and β-carotene (19% on average) (Fig. 2A). The peels of a further seven red-fleshed and seven white-fleshed loquat cultivars at ripe stages were also analysed, and contained on average 19.1 μg g^–1^ FW carotenoids in the white-fleshed cultivars, which was ~25% of the values in the peel of red-fleshed cultivars (Supplementary Fig. S2; Supplementary Table S7 at *JXB* online).

The colour of LYQ and BS leaves appeared similar (Fig. 2). Although the carotenoid colour was masked by chlorophylls, there was no photobleaching of leaves, which might have been expected if the carotenoid content had been low. To determine whether the carotenoid accumulation patterns in BS leaf were affected, the content in young and mature leaves of LYQ and BS was analysed. This comparison showed that young and mature BS leaves contained an ~20% lower amount of carotenoids as compared with LYQ (Fig. 2C; Supplementary Table 8 at *JXB* online). The leaf carotenoid profiles, however, were very similar between LYQ and BS loquats.

### Isolation of four *PSY* genes from loquat

**Fig. 3.**
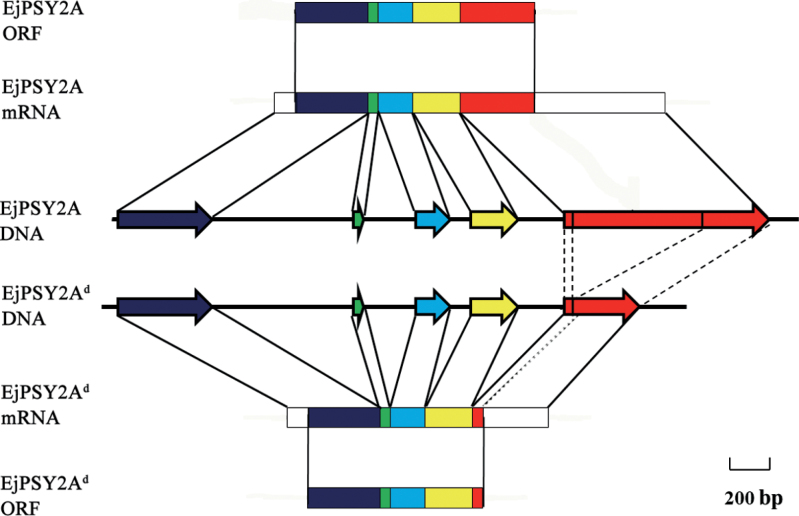
The structure of *EjPSY2A* and *EjPSY2A* ^*d*^. The *EjPSY2A* ^*d*^ sequence is mutated, missing a 694bp nucleotide sequence in the fifth exon of the *EjPSY2A* gene. The dark blue region is the first exon, the green region is the second exon, the blue region is the third exon, the yellow region is the fourth exon, and the red region is the fifth exon. Thin lines indicate introns. The coloured boxes show the translated sequence and the white boxes the untranslated segments.

Three unigenes (Unigene13461, Unigene46522, and Unigene26970) were found in the LYQ library and one, Unigene 54535, was identified in the BS library. When the complete sequences of these unigenes were obtained by RACE, Unigene13461 was named *EjPSY1*; Unigene46522 was named *EjPSY2A*; Unigene26970, which had high homology with Unigene46522, was named *EjPSY2B*; and Unigene54535 in BS was 321 bases shorter than the full length of *EjPSY2A* and was designated *EjPSY2A* ^*d*^. Upon sequencing, the cDNA of *EjPSY2A* ^*d*^ was found to be identical to that of *EjPSY2A* for the first 861 nucleotides of translated sequence. Thereafter, homology broke down completely for a further 321 nucleotides until the 3′-untranslated region (UTR) sequence was reached. An in-frame stop codon exists within this 321 base sequence, leading to a predicted protein of 33kDa, compared with 45.1kDa for the normal sequence. The first 287 residues of this 290 amino acid truncated sequence were shared with the 397 amino acids encoded by the LYQ *EjPSY2A* (Supplementary Fig. S3 at *JXB* online). Further, the genomic DNA of the mutant *EjPSY2A* ^*d*^ showed that the DNA sequence homology broke down in the fifth exon, resulting in the mutant mRNA of *EjPSY2A* ^*d*^ (Fig. 3). Degenerate primers (Supplementary Table S3 at *JXB* online) were designed according to an additional and distinct plant *PSY* deposited in the Phytozome database (http://www.phytozome.net/), and, following PCR, an additional loquat family member, *EjPSY3*, was obtained. Sequencing of the *EjPSY3* mRNAs found in LYQ and BS varieties showed the occurrence of different sequences that appeared to be due to altered RNA splicing. *EjPSY3* in the LYQ variety (named *EjPSY3α*) lacked 97bp of the fourth exon, whereas the *EjPSY3* from the BS variety (named *EjPSY3β*) lacked 10bp of the third exon and contained the third intron, and both sequences displayed several stop codons (Supplementary Fig. S4). When other loquat varieties were tested, a normal *EjPSY3* was also observed, for example in Jiajiao (red-fleshed), Baiyu, and Biqi (white fleshed).

**Fig. 4.**
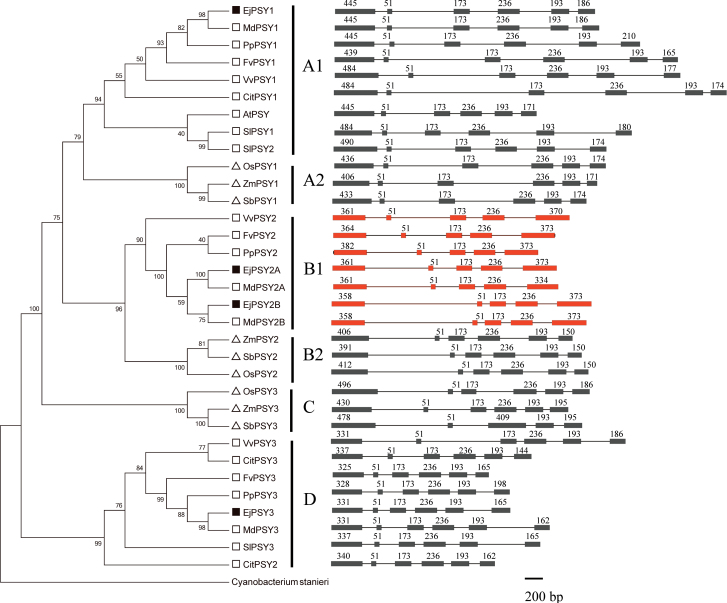
Phylogenetic tree (left) and the DNA structure (right) of PSYs. Phylogenetic tree: dicot plants are indicated by open squares, monocot plants by triangles, and loquat genes by filled squares. Amino acid sequences were aligned using ClustalW and a Neighbor–Joining tree was constructed with a 1000-bootstap replication support using MEGA5 software. DNA structure: boxes and thin bars indicate exons and introns, respectively; The DNAs having five exons are indicated with red boxes and those with six exons with black boxes.

The deduced amino acid sequences for EjPSY1, EjPSY2A, EjPSY2B, and EjPSY3 were determined and aligned (Supplementary Fig. S5 at *JXB* online). What most distinguished EjPSY1, EjPSY2A, EjPSY2B, and EjPSY3 sequences from each other were the N-terminal regions. To facilitate a more in-depth analysis of the four loquat *EjPSY* gene family members, the genomic DNA sequences were obtained and the deduced amino acid sequences were used for phylogenetic analysis (Fig. 4). *EjPSY1* and *EjPSY3* genes possess six exons and five introns, and differ from *EjPSY2A* and *EjPSY2B*, which have five exons and four introns. Phylogenetic analysis showed that *PSY* genes could be divided into four groups, here called A (divided into A1 and A2), B (divided into B1 and B2), C and D. *EjPSY1* belongs to group A1, *EjPSY2A* and *EjPSY2B* together belong to group B1, and *EjPSY3* clustered in another group, D. Notably, the *PSY* genes in group D are the most closely related to *CrtB* of *Cyanobacterium*. For comparison, the three *PSY* family members in the Poaceae (*PSY1*-like, *PSY2*-like, and *PSY3*-like) were clustered in groups A2, B2, and C, respectively.

### Reverse transcription–PCR analysis of expression of all *EjPSYs* transcripts in LYQ and BS tissues

If *EjPSY1*, *EjPSY2A*, *EjPSY2B*, or *EjPSY3* mRNA expression correlates with carotenoid accumulation, it would be expected that their transcripts would vary specifically in tissues of peel, flesh, leaf, or root, given that peel and leaf of the BS variety accumulate carotenoids and the flesh does not. To test this possibility, the relative expression of *EjPSY1*, *EjPSY2A* (in LYQ), *EjPSY2A* ^*d*^ (in BS), *EjPSY2B*, *EjPSY3α* (in LYQ), and *EjPSY3β* (in BS) transcript levels were estimated in RNA samples derived from root, stem, petal, young and mature leaf, peel, and flesh at three fruit developmental stages (mature green, breaker, and ripe) using quantitative RT–PCR with gene-specific primers.

**Fig. 5.**
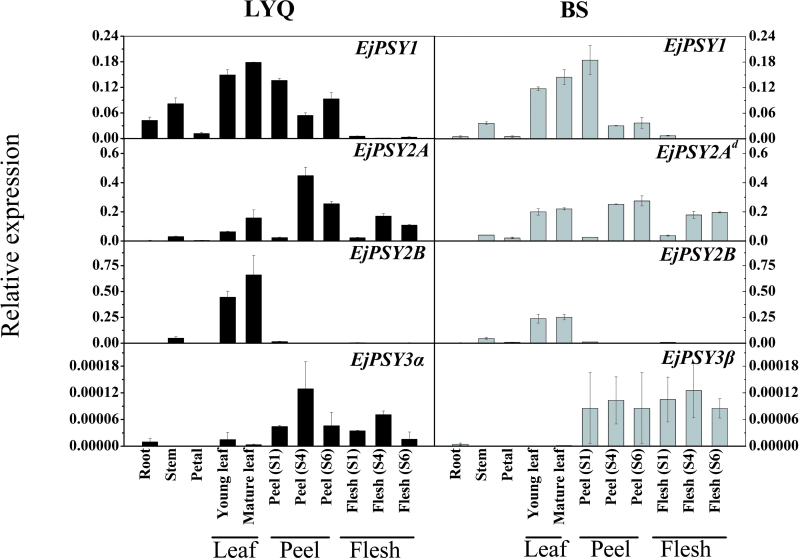
mRNA levels of *EjPSY1, EjPSY2A* (LYQ), *EjPSY2A* ^*d*^ (BS), *EjPSY2B*, *EjPSY3α* (LYQ), and *EjPSY3β* (BS) in different tissues. The data show mRNA levels relative to actin mRNA. Note the differences in the scales for different genes. Loquat tissues examined: root, stem, petal, young leaf and mature leaf, peel at the green stage (S1), peel at the breaker stage (S4), peel at the ripe stage (S6), flesh at the green stage (S1), flesh at the breaker stage (S4), and flesh at the ripe stage (S6).

**Fig. 6.**
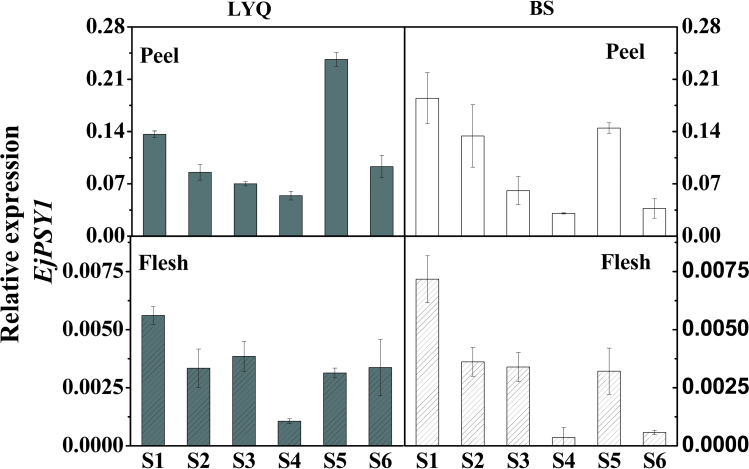
The expression patterns of the *EjPSY1* gene in peel and flesh of LYQ and BS during fruit ripening.

The *EjPSY1* transcripts could be detected in almost all the tissues examined and were highest in leaf, stem, and peel, but were present at extremely low levels in petal and mature fruit flesh (Fig. 5). In the flesh tissue, *EjPSY1* transcripts were always 15-fold lower than those in the peel during all stages of fruit development in both LYQ and BS (Fig. 6). *EjPSY2A* (LYQ) transcripts were present at very low levels in the tissues of root, stem, and petal, and in the peel and flesh of fruit at the mature green stage. As expected, however, the *EjPSY2A* (LYQ) transcript showed a typical increase between the mature green and breaker stage in fruit peel, with a slight reduction between the breaker and ripe stages. The *EjPSY2A* ^*d*^ transcript levels in BS were not affected by the sequence mutation, and showed a similar expression pattern and level to *EjPSY2A* in LYQ (Fig. 5).

Accumulation of the *EjPSY2B* mRNA occurred to the highest level in leaf tissue, and the content was a little higher in LYQ leaves compared with those of BS. The transcripts of *EjPSY2B* in stem, peel, and flesh at the mature green stage were present at higher concentrations than in the tissues of root, petal, peel, and flesh at breaker and ripe stages, although they were still very low compared with the content in leaf RNA (Fig. 5). This is consistent with the EjPSY2B enzyme playing a pivotal role in providing carotenoids for the assembly of the photosynthetic apparatus in leaves and other green tissues. The *EjPSY3α* and *EjPSY3β* transcripts showed similar expression patterns in LYQ and BS and had the lowest expression levels of all the *EjPSY* family members in loquat, at least 1000-fold less than other members, and were sometimes undetectable by quantitative PCR (Fig. 5). Very few *EjPSY3* transcripts were detected by quantitative PCR in the flesh of Jiajiao, Baiyu, or Biqi cultivars, which was similar to the situation for *EjPSY3α* in LYQ and *EjPSY3β* in BS cultivars.

### Functional analysis of all EjPSYs in loquat

**Fig. 7.**
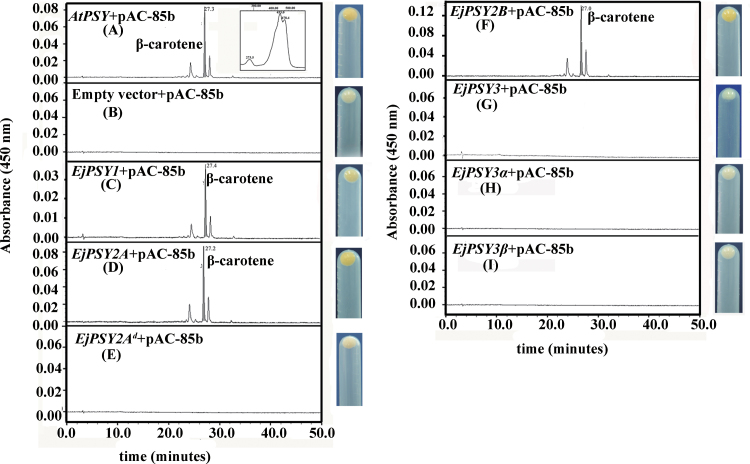
Effectiveness of *EjPSY1*, *EjPSY2A*, *EjPSY2A* ^*d*^, *EjPSY2B*, *EjPSY3*, *EjPSY3α*, and *EjPSY3β* from loquat in functional complementaion of carotenoid synthesis in *E. coli*. *Escherichia coli* cells were co-transformed with *AtPSY*+pAC-85b (A); empty vector+pAC-85b (B); *EjPSY1*+pAC-85b (C); *EjPSY2A*+pAC-85b (D); *EjPSY2A* ^*d*^+pAC-85b (E); *EjPSY2B*+pAC-85b (F); *EjPSY3*+pAC-85b (G); *EjPSY3α*+pAC-85b (H); and *EjPSY3β*+pAC-85b (I). Chromatograms show HPLC separation of extracted pigments; the inset in (A) shows the spectral fine structure for the pathway end-product, β-carotene. The pellet colours of the cells are shown on the right.

To determine whether the EjPSY members EjPSY1, EjPSY2A, EjPSY2A^d^, EjPSY2B, EjPSY3, EjPSY3α, and EjPSY3β are functional in a bacterial carotenoid synthesis system, the ORF of each corresponding gene was subcloned into the *E. coli* expression vector and co-transformed along with the pAC-85b plasmid, which contains all coding sequences necessary for carotenoid biosynthesis, except PSY. The expected product, β-carotene, confirmed by matching spectra and column chromatography retention times, as well as two other carotenoids, probably β-carotene isomers according to their spectra, were produced in the bacteria transformed with the EjPSY1, EjPSY2A, and EjPSY2B vectors, while no carotenoids were detected with EjPSY3, the mutant EjPSY2A^d^, as well as vectors carrying the mis-spliced EjPSY3α and EjPSY3β sequences (Supplementary Fig. S4 at *JXB* online). This indicates that the *EjPSY1*, *EjPSY2A*, and *EjPSY2B* cDNAs all encoded enzymes that were functional in the bacterial system. In contrast, the mutant *EjPSY2A* ^*d*^ found in BS was non-functional, presumably due to the loss of the C-terminal region (Figs 3, 7), and the mis-spliced *EjPSY3α* found in LYQ and *EjPSY3β* found in BS were also non-functional. Unexpectedly, *EjPSY3* was probably also non-functional although it encodes a full-length translatable sequence (Fig. 7).

### The phenotype of white-fleshed loquat is determined by the recessive gene *EjPSY2A* ^*d*^

**Fig. 8.**
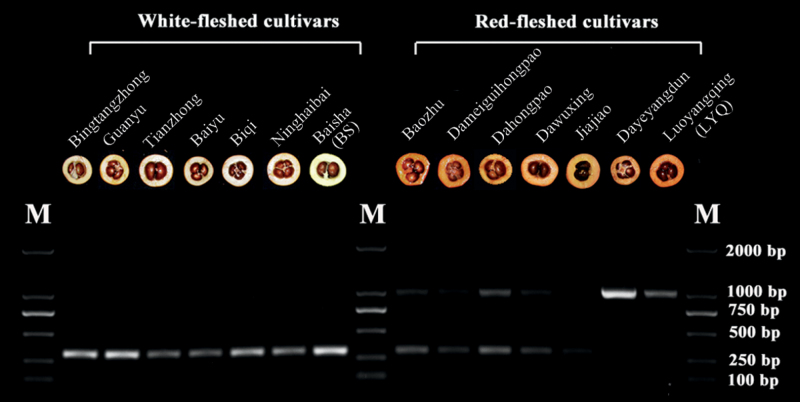
PCR amplification of the *EjPSY2A*/*EjPSY2A* ^*d*^ genomic region from red- and white-fleshed loquat varieties. PCR amplification of the *EjPSY2A*/*EjPSY2A* ^*d*^ genomic region gave two fragments: a 1013bp fragment (*EjPSY2A*) present only in red-fleshed varieties and absent in white-fleshed varieties, and a 319bp fragments (*EjPSY2A* ^*d*^) present in white-fleshed types and some red-fleshed cultivars, together with the 1013bp fragment.

**Fig. 9.**
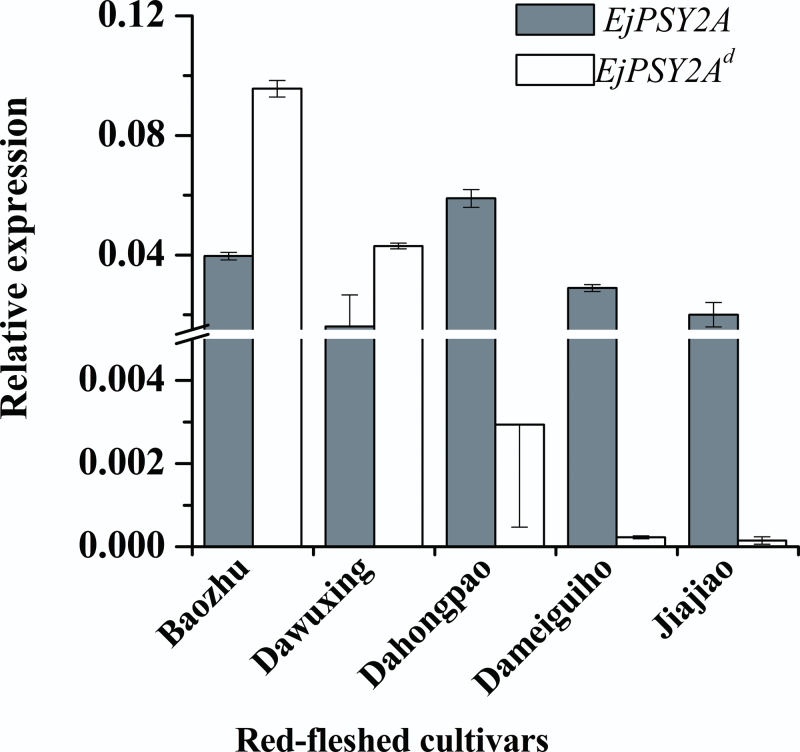
The *EjPSY2A* and *EjPSY2A* ^*d*^ mRNA expression levels in flesh of five ripe red-fleshed fruit cultivars. The red-fleshed cultivars Baozhu, Dawuxing, Dahongpao, Dameiguihongpao, and Jiajiao contained the DNA sequence of the normal *EjPSY2A* gene and the mutant *EjPSY2A* ^*d*^.

The differences in the expression level of carotenogenic genes between LYQ (red-fleshed) and BS (white-fleshed) loquat seemed insufficient to explain the large differences in carotenoid content between the two cultivars (Fu *et al.*, 2012). Here, the mutant *EjPSY2A* ^*d*^ cloned from the BS (white-fleshed) variety showed specific expression in mature fruit (Fig. 7), although it was truncated and had no PSY catalytic activity in the bacterial system (Fig. 7), indicating that this mutation is likely to be responsible for the white flesh colour of BS. The flesh of the LYQ (red-fleshed) variety, expressing *EjPSY2A*, did, on the other hand, accumulate abundant carotenoids. There are several other red- and white-fleshed loquat cultivars cropped in China. To investigate whether the mutant *EjPSY2A* ^*d*^ occurred in other white-fleshed loquat or also existed in other red-fleshed loquat cultivars, a specific primer pair was designed across the region of the lost sequence in *EjPSY2A* ^*d*^, so that only one short band (319bp) could be amplified if the cultivar contained only the mutant sequence *EjPSY2A* ^*d*^, whereas only one larger band (1013bp) would be generated if the cultivar had only the normal sequence *EjPSY2A*. The production of both bands would indicate the presence of both *EjPSY2A* and *EjPSY2A* ^*d*^ in the cultivar. The results clearly showed (Fig. 8) that all the white-fleshed loquat cultivars tested possessed only the same mutant *EjPSY2A* ^*d*^ gene structure, and all the red-fleshed loquat cultivars contained the *EjPSY2A* sequence encoding the functional enzyme. Interestingly, some red-fleshed loquat cultivars (Baozhu, Dawuxing, Dahongpao, Dameiguihongpao, and Jiajiao) also contained the mutant *EjPSY2A* ^*d*^ gene (Fig. 8). In addition, the mutant *EjPSY2A* ^*d*^ mRNA also had high expression levels in the flesh of Baozhu and Dawuxing at the mature stage (Fig. 9), indicating that the white flesh of loquat was controlled by this recessive gene, and that some red-fleshed varieties were heterozygous for this gene.

## Discussion

### *EjPSY2A* plays the main role in flesh carotenoid biosynthesis in mature fruit of loquat

Red-fleshed loquat varieties all contain a functional *EjPSY2A* (Figs 7, 8), which was highly expressed in fruit flesh and was probably responsible for carotenoid accumulation in the flesh (Fig. 5). A mutated non-functional variant of this gene (*EjPSY2A* ^*d*^) was detected in all the white-fleshed loquat cultivars examined (Fig. 8). Thus, although *EjPSY2A* ^*d*^ is expressed in the flesh of white-fleshed cultivars, the non-functional enzyme (Fig. 7) it encodes is unable to participate in carotenoid biosynthesis, showing a convincing correlation with the greatly reduced level of carotenoids accumulated in the flesh of white-fleshed loquat cultivars. This phenomenon is similar to the situation in the yellow-fleshed tomato *r* mutant, which possessess a non-functional *PSY1* gene and does not accumulate carotenoids in the fruit (Fray and Grierson, 1993). Transfer of the *EjPSY2A* gene to white-fleshed cultivars would be a direct way to validate further whether the failure of carotenoid accumulation in flesh resulted solely from the loss-of-function mutation of EjPSY2A; however, at present, a genetic transformation system is not available for loquat, although such a system has been successfully used for several other Rosaceae plants (Espley *et al.*, 2007; Gambino and Gribaudo, 2012).

It is interesting that some red-fleshed loquat cultivars possess both the functional enzyme EjPSY2A and the non-functional mutant EjPSY2A^d^, and high transcript levels of the inactive EjPSY2A^d^ were present in the flesh of some red-fleshed cultivars. However, this heterozygosity of *EjPSY2A* and *EjPSY2A* ^*d*^ in the red-fleshed loquat cultivars did not affect the overall carotenoid accumulation (Fig. 1), which is entirely consistent with the phenotype of the white-fleshed loquat cultivars being controlled by the recessive gene *EjPSY2A* ^*d*^. According to the analysis of different cultivars, the first white-fleshed loquat cultivar might have arisen from one of the red-fleshed cultivars by mutation, followed by segregation. Red-fleshed loquat cultivars heterozygous for *EjPSY2A* and *EjPSY2A* ^*d*^ might have descended from trees heterozygous for the mutation, or, alternatively, might have arisen by hybridization between red-fleshed and white-fleshed cultivars.

In a previous study, no chromoplast structure was found in the cells of ripe BS fruit flesh (Fu *et al.*, 2012); it would be interesting to investigate in future studies whether the failure of chromoplast development in BS flesh can be due to the non-functional mutant EjPSY2A^d^. There is evidence from other studies that plastid morphology can be affected by elevated carotenoid levels in canola endosperm (Shewmaker *et al.*, 1999), and the expression of *PSY* was tightly related to chromoplast formation (Fraser *et al.*, 2007; Nogueira *et al.*, 2013; Bai *et al.*, 2014). The mutation of critical amino acid residues in maize PSY can result in changes in localization and cause distorted plastid shape and formation of a fibril phenotype (Shumskaya *et al.*, 2012), and cellular structures can be accordingly adapted to facilitate the sequestration of newly formed products (Nogueira *et al.*, 2013).

### The expression of *EjPSY1* and *EjPSY2B* is responsible for accumulation of carotenoids in the peel and leaf of white-fleshed loquat

The tissue-specific expression of *EjPSY1*, *EjPSY2A*, *EjPSY2B*, and *EjPSY3* revealed unique features of the four gene family members. Transcripts of *EjPSY2A* were in low abundance in root and petal, detectable in stem, leaf, and green fruits (peel and flesh), and most abundant in breaker fruits. The role and importance of catalytically active *EjPSY2A* in loquat fruit carotenoid accumulation was established by the lack of carotenoids accumulated in the flesh of white-fleshed loquat expressing the catalytically inactive *EjPSY2A* ^*d*^ mutant. In contrast to *EjPSY2A*, *EjPSY2B* transcripts were only abundant in leaf tissue, and transcripts were present in only very low amounts in other organs and tissues, which supports the proposal that *EjPSY2B* is responsible for carotenoid production in green tissues, especially in the leaf. This explains why young fruits and leaves of the white-fleshed loquat cultivars with the non-functional *EjPSY2A* ^*d*^ can still accumulate normal amounts of carotenoids, because *EjPSY2B* plays the main role in carotenoid accumulation in the young fruit and leaf of both red- and white-fleshed varieties.

According to the expression pattern, *EjPSY1* was present in all tissues, with few transcripts in fruit flesh but with a higher expression level in the peel in both LYQ and BS loquat fruits. The observation that *EjPSY1* had higher expression in the peel explains very well how the peel of white-fleshed cultivars can accumulate moderate amount of carotenoids during ripening (Fig. 6). Differential carotenoid accumulation patterns have also been discovered in tissues of the Cara Cara citrus fruit, where the flesh accumulates large amounts of lycopene, but the peel tissue does not (Xu *et al.*, 2006), although the underlying mechanism is not yet known.

### The function of EjPSY3 remains to be determined

*EjPSY3* showed the lowest mRNA accumulation in all tissues examined, and different RNA editing mutations occurred in the LYQ and BS varieties, resulting in the production of non-functional enzymes (Figs 5, 7), although the normal cDNA of *EjPSY3* was observed in some other loquat varieties such as Jiajiao, Baiyu, and Biqi. However, the normal sequence of *EjPSY3* also lacked catalytic activity in the *E. coli* system. This might be due to several amino acid differences, compared with other functional *EjPSY* genes (Supplementary Figs S5, S6 at *JXB* online), in or near the normally conserved domain 2 (Shumskaya *et al.*, 2012). There have been several reports that a single amino acid change can alter the activity of a PSY enzyme. Welsch *et al.* (2010) showed that a single nucleotide polymorphism in *PSY2*, causing a non-conservative amino acid exchange, leads to markedly increased carotenoid formation and accumulation in cassava storage roots. Gady *et al.* (2012) indicated that the P192L mutation (an amino acid substitution P192L) affects PSY1 activity through misfolding, leading to low phytoene accumulation.

Due to the lack of catalytic activity and much lower expression level of PSY3, it seems not to play a role in carotenoid accumulation in the loquat plant. Different splicing mutations were found in LYQ and BS, which showed there were variations in mRNA processing, although the significance of this is not clear. EjPSY3 might possibly have had a role previously, but lost its catalytic activity during the course of evolution. The amino acid sequence of EjPSY3 was most similar to that of SlPSY3, which has also been reported to be the least expressed *PSY* gene in tomato. SlPSY3 does not appear to play a major role in fruit lycopene biosynthesis, although when *PSY3* was silenced in tomato fruit a small but significant reduction of phytoene, phytofluene, γ-carotene, and δ-carotene was observed (Fantini *et al.*, 2013). However, the catalytic activity of SlPSY3 has not yet been examined. The *PSY3* family members from dicot plant shared high homology (Fig. 4) and clustered in group D. A striking difference was found in the highly conserved coding region 2 (Supplementary Fig. S6 at *JXB* online) of PSY3, at amino acid residue 264, which was glycine (Gly264) in dicot plants, compared with alanine in other plant PSYs, including CrtB. Furthermore, the nearby position, 260, was occupied by tyrosine, asparagine, or histidine in dicot plant PSY3s (Tyr260, Asn260, or His260), in contrast to the alanine present in all other PSYs, also including CrtB. Whether these amino acid differences affect the function of EjPSY3s requires further study, and the properties of *PSY3* genes in other dicots (such as tomato or citrus) require further investigation to test whether they are also catalytically inactive, or whether they have retained activity.

### *PSY* expression patterns are independent of gene structure and evolution

Only one phytoene synthase gene family member has been found in *Arabidopsis thaliana* (Lange and Ghassemian, 2003), and three family members are found in *Solanum lycopersicum* (Tomato Genome Consortium, 2012). In this study, four family members were found, and the function, evolution, and structure of these genes were analysed. According to the phylogenetic tree, *EjPSY3* was clustered with *SlPSY3* in group D, which is considered much more ancient than other plant phytoene synthase genes. On the basis of the present evidence, it seems likely that *EjPSY1*-type genes (six exons) in group A1 possibly evolved from *PSY3* genes in group D. *EjPSY2A*–*EjPSY2B-*type genes (five exons) in group B1 of some dicot plants (e.g. *Eriobotrya japonica*, *Vitis vinifera*, and *Prunus persica*) probably arose by the fusion of the fifth and sixth exons during evolution.

The *PSY* evolutionary branches in dicot plants seem quite diverse. For example, one branch has either been lost (*A. thaliana*, which only has one gene), or has lost catalytic activity (EjPSY3 in group D). Also, where duplication in one of the three branches has taken place (e.g. *SlPSY1* and *SlPSY2*), a new *PSY* DNA structure (*EjPSY2A* and *EjPSY2B*) has developed during the course of evolution.

Although the gene structures and evolutionary relationships (Fig. 4) are different among species, the expression patterns of these genes are conserved. For example, the structures of *SlPSY1* and *SlPSY2* in tomato (expressed in fruit and leaves, respectively, clustered in group A1, with six exons and five introns) are different from those of *EjPSY2A* and *EjPSY2B* in loquat (clustered in group B1, with five exons and four introns), but they play similar roles in carotenoid synthesis in the respective plants (Figs 4, 5; Fraser *et al.*, 1999). Paradoxically, according to the gene structures, although *EjPSY1* is closer to *SlPSY1* and *SlPSY2*, clustered in group A1, all with six exons and five introns, the expression patterns are different, with *EjPSY1* having few transcripts in flesh. This strongly suggests that different *PSY* genes have been recruited to perform similar roles during the evolution of different fruits.

### GenBank accession numbers

Sequence data from this article have been deposited in the EMBL/GenBank data libraries under accession numbers: *EjPSY1* (KF922363), *EjPSY2A* (KF922364), *EjPSY2A* ^*d*^ (KF922365), *EjPSY2B* (KF922366), *EjPSY3* (KF922367), *EjPSY3α* (KF922368), and *EjPSY3β* (KF922369).

## Supplementary data

Supplementary data are available at *JXB* online.

Figure S1. Unigene sequence of PSY in loquat RNA-Seq libraries.

Figure S2. Carotenoid content and composition in the peel of red- and white-fleshed loquat cultivars.

Figure S3. Comparison of the deduced amino acid sequences of EjPSY2A and EjPSY2A^d^.

Figure S4. Analysis of the alternative splicing of EjPSY3α and EjPSY3β.

Figure S5. Alignment of EjPSY amino acid sequences.

Figure S6. Alignment of PSY amino acid sequences.

Table S1. Primers for RACE PCR.

Table S2. Primer sequences for genome walking.

Table S3. Primers for genomic DNA PCR.

Table S4. Primer sequences for PCR amplification of the *EjPSY2A*/*EjPSY2A* ^*d*^ genomic fragment.

Table S5. Primers for real-time PCR.

Table S6. Primers for PSY cDNAs subcloned as in-frame translational fusions.

Table S7. Carotenoid content in peel and flesh tissues of white- and red-fleshed loquat cultivars.

Table S8. Carotenoid content in various tissues of LYQ and BS.

Supplementary Data
