# Supplementary material for: Involvement of multiple phytoene synthase genes in tissue- and cultivar-specific accumulation of carotenoids in loquat
Source: J Exp Bot. 2014 Jun 16;65(16):4679–89. doi: 10.1093/jxb/eru257 (PMC4115255; doi:10.1093/jxb/eru257)
Supplement: Supplementary Data [file supp_65_16_4679__index.html]

Involvement of multiple phytoene synthase genes in tissue- and cultivar-specific accumulation of carotenoids in loquat — Involvement of multiple phytoene synthase genes in tissue- and cultivar-specific accumulation of carotenoids in loquat — Supplementary Data 

# Involvement of multiple phytoene synthase genes in tissue- and cultivar-specific accumulation of carotenoids in loquat

## Supplementary Data

Data files

**Files in this Data Supplement:**

- Supplementary Data - Supplementary Data
