# Supplementary material for: Involvement of multiple phytoene synthase genes in tissue- and cultivar-specific accumulation of carotenoids in loquat
Source: J Exp Bot. 2014 Jun 16;65(16):4679–89. doi: 10.1093/jxb/eru257 (PMC4115255; doi:10.1093/jxb/eru257)
Supplement: Supplementary Data [file supp_eru257_jexbot123729_file001.pdf]

Involvement of multiple phytoene synthase genes in tissue- and cultivar-specific accumulation of carotenoids in loquat

Xiumin Fu, Chao Feng, Chunyan Wang, Xueren Yin, Pengjun Lu, Don Grierson, Changjie Xu, Kunsong Chen

>Unigene46522_LYQ
ATTTGCCAACTACCACTGCTTTCAAAACTTTTGCCTTACCCTCCAATCTCCATAAGAGAAGCCACAACCTCACAGCTCACATACT
CACACCCTCACACTCAAAACCTAAAACCCAACAGATTAATTGTTCTTTTGATATGTCAGTTGTTCTTCTTTGGGTGGTGAGCCCC
AAACAGAATGCCAGCTCCCTGCTGGGTCTGATGCCCAGAATTTGCACCCCAAGGAGGTCTAAATTCTGCCCGAAGCTGGGATTT
TCAAGTAGGGTTTTGGCCTACTCGGGTGCAGTTGTAAACCCAGCAAGGTCTTCAGAAGAGAAGGTGTATGAAGTGGTGCTGAA
GCAGGCTGCGCTGGTGGAAGAACAGAGCACGGTAAAAAGGAAATCTTTGGATTTGGATGAACGGATTGTAACTGAAGGTTTGG
ACAACTGGGATTTACTGGATAAGGCTTATGATCGGTGTGGTGAAGTCTGTGCTGAGTATGCCAAGACTTTTTACCTGGGCACAT
TGCTCATGACACCGGAGCGGCGACGAGCTGTTTGGGCAATCTATGTGTGGTGCAGAAGGACAGATGAGCTAGTGGATGGACCT
AATGCTTCATACATTACACCCAAAGCTCTTGACAGATGGGAGAAAAGACTGACTGATCTCTTTGAAGGCCGACCTTACGATATG
TATGATGCTGCTCTATCTGATACTGTCACCAAGTACCCCGTTGACATTCAGCCCTTCAGAGACATGGTAGAAGGAATGAGATTA
GACTTGAGGAAGTCAAGATACCAGAACTTTGATGAACTTTACCTTTACTGCTACTATGTTGCTGGAACTGTTGGATTGATGAGT
GTTCCGGTGATGGGGATATGCCCAGAATCAAGGGCCTCAACAGAAAGCGTTTACAATGCTGCATTGGCCCTTGGAATTGCTAAT
CAGCTCACTAACATTCTCAGAGATGTTGGAGAAGATGCTAGGAGAGGAAGAGTATATCTCCCCCAAGATGAGCTTGCCCAAGC
CGGCCTATCAGACGATGACATCTTTCGCGGGAAGGTGACTGACAAGTGGCAAAGTTTCATGAAGGGACAAATACAGAGAGCTA
GGATGTTCTTTGATGAGGCTGAGAAGGGTGTCTCAGAGCTCAACTCAGCTAGTAGATGGCCAGTATGGGCATCTTTGTTGCTGT
ATAGGCAGATTCTAGATGTAATTGAAGCAAATGGTTATGACAATTTCACAAAAAGGGCTTATGTGGGAAAAGCAAAGAAGTTT
GTATCGTTGCCTGTGGCCTATGGAAGAGCCATTATAGGACCCTCTAAATTAACTAAGCAGTTGGTGCCTAGATGAATTAGATGA
TATTTGAAGTCTCAATCTCTAAATTTTTAGACAAAGTCCAACTTGAAGTTGGGTCTCAAGTCGAAATATGTAAATTTTTTATACT
AAATTTTTTTTAGTCAATCTTTTAATTACCTGTGTTTGGATGACGGATATAAAAGTGGTAATTAGTGGCAGAGGTTAATAGATGT
GTGGTGAGAGTGGTCGCCATGGACTGGTTGATAGATGTGTCAGTGGAGGTAGAGTGTCTCCTAGTGATAAGGTGGTAGCAATT
GTGGTTGTGGCATTGGCGGTGGTGATGGTTGTGGGAGGTGGTGGTGACGGTGATGGTGGATATGATTGGAATGATGGTGGCAA
TAGCAATGACATTGGGGGTTCATGGTAGTACTTATGGTCATGAGAGT
>Unigene51742_LYQ
TGTCTAAAAATTTAGAGATTGAGACTTCAAATATCATCTAATTCATCTAGGCACCAACTGCTTAGTTAATTTAGAGGGTCCTAT
AATGGCTCTTCCATAGGCCACAGGCAACGATACAAACTTCTTTGCTTTTCCCACATAAGCCCTTTTTGTGAAATTGTCATAACCA
TTTGCTTCAATTACATCTAGAATCTGCCTATACAGCAACAAAGATGCCCATACTGGCCATCTACTAGCTGAGTTGAGCTCTGAG
ACACCCTTCTCAGCCTCATCAAAGAACATCCTAGCTCTCTGTATTTGTCCCTTCATGAAACTTTGCCACTTGTCAGTCACCTTCC
CGCGAAAGATGTCATCGTCTGATAGGCCGGCTTGGGCAAGCTCATCTTGGGGGAGATATACTCTTCCTCTCCTAGCATCTTCTC
CAACATCTCTGAGAATGTTAGTGAGCTGATTAGCAATTCCAAGGGCCAATGCAGCATTGTAAACGCTTTCTGTTGAGGCCCTTG
ATTCTGGGCATATCCCCATCACCGGAACACTCATCAATCCAACAGTTCCAGCAACATAGTAGCAGTAAAGGTAAAGTTCATCA
AAGTTCTGGTATCTTGACTTCCTCAAGTCTAATCTCATTCCTTCTACCATGTCTCTGAAGGGCTGAATGTCAAC
>Unigene26970_LYQ
CGTCGCCGCTCCGGTGTCATGAGCAATGTCCCTAGGTAAAAAGTCTTGGCATACTCTGCACAGACCTCACCACACCGGTCATAC
GCCTTATTCAGTAAATCCCAGTTGTTCAAACCGTCAGTAATCCGTTCATCCAAATCCAAAGATTTCTTTTTTACCGTGTTCGGTT
CTCTCACTAGAGCAGCCTGCTTCA
>Unigene32141_LYQ
CGTCGCCGCTCCGGTGTCATGAGCAATGTGCCCAGGTAAAAAGTCTTGGCATACTCAGCACAGACTTCACCACACCGATCATAA
GCCTTATCCAGTAAATCCCAGTTGTCCAAACCTTCAGTTACAATCCGTTCATCCAAATCCAAAGATTTCCTTTTTACCGTGCTCT
GTTCTTCCACCAGCGCAGCCTGCTTCAGCACCACTTCATACACCTTCTC

>Unigene38534_LYQ
TACTCACACCCTCACACTCAAAACCTAAAACCCAACAGATTAATTGTTCTTTTGATATGTCAGTTGTTCTTCTTTGGGTGGTGAG
CCCCAAACAGAATGCCAGCTCCCTGCTGGGTCTGATGCCCAGAATTTGCACCCCAAGGAGGTCTAAATTCTGCCCGAAGCTGG
GATTTTCAAGTAGGGTTTTGGCCTACTCGGGTGCAGTTGTAAACCCAGCAAGGTCTTCAGAAGAGAAGGTGTATGAAGTGGTG
CTGAAGCA
>Unigene7419_LYQ
CTTCTCTGGGTGGTGAGTCCCAAAGAGAATGCCAACTCTCTGCTGGGTCTGTTGCCGAGAATTTGCACCCCAAGGAGGTCTAAA
TTGTGCTCAAAGCTGGGTTTTTCAAGTGGNNNNNNNNNNNNNNNNNNNNNNNNNNNNNNNNNNNCAGTTGCAAACCCAGCCAG
ATCTTCAGAGGAGAAGGTGTATGAAGTGGTGCTGAAGCA
>Unigene7419_LYQ
CTTCTCTGGGTGGTGAGTCCCAAAGAGAATGCCAACTCTCTGCTGGGTCTGTTGCCGAGAATTTGCACCCCAAGGAGGTCTAAA
TTGTGCTCAAAGCTGGGTTTTTCAAGTGGNNNNNNNNNNNNNNNNNNNNNNNNNNNNNNNNNNNCAGTTGCAAACCCAGCCAG
ATCTTCAGAGGAGAAGGTGTATGAAGTGGTGCTGAAGCA
>Unigene13461_LYQ
AGATACCAAAGCTTCGATGAACTATATCTTTACTGCTATTATGTTGCCGGAACTGTTGGATTAATGAGTGTTCCAGTTATGGGC
ATTTCACCTGAATCGCAAGCAACAACGGAGAGTGTATATAACGCTGCCTTGGCGTTAGGGATTGCGAATCAGCTAACAAACAT
ACTCCGTGATGTTGGGGAGGATGCAAGGAGAGGAAGAATTTATTTACCCCAAGATGAGCTTGCAGAAGCAGGGCTCTCCGACG
CTGATATATATGCTGGGAAGGTCACAGACAAATGGAGGAGTTTCATGAAGGATCAAATTAAGAGGGCAAGGATGTTCTTCGAC
GAGGCAGAGAAGGGAGTGACAGAGTTGAGTGAAGCCAGCAGATGGCCGGTATTGGCGTCGCTACTCCTATATCGCCAAATATT
GGACGAGATAGAAGCTAACGATTACNNNNNNNNNNNNNNNNNNNGTAAGCAAAGCTAAGAAATTACTTGCCTTGCCTATTGCG
TATACTAAATCGATAATTCGCCCCCCAAGAACCTCTCCAGAGCTTAGAAAATACAATCTTTGACAACAAAATGCTGGAGTTGCC
TATATATGTATAGANNNNNNNNNTGTATAGAGATTGAAAAGCAGAATTTGTATACTATAAATTGTATTGTTGCAGCCTGTATTTA
TAAAACTGAAGGGTGTTATTTATTCCAGTTCAAAG
>Unigene46044_LYQ
GGAGGGTCTGGTTTAGGGAGCGAAACCAAATTTCCAGTTTTATTAAGCATGGTGGCCAACCCGCTTGGAGAATCAGCTGTCTCG
TCGGAACAGAAGGTGTATGATGTGGTGCTGAAGCAGGCATCCTTGGGTAAGAAGCAGTTGAGTTCTAATGGATATCTTGATGT
GAAGCGGGATATTATTCTACCCGGGAATCTGAGCTTGTTGAGTAAAGCTTATGATCGATGTGGAGAAGTATGTGCGGAGTATG
CCAAGACCTTTTACCTCGGTACCCTGCTTATGACGCCCGAAAGAAGAAGGGCCATCTGGGCAATATATGTGTGGTGCAGGAGG
ACGGATGAGCTTGTTGATGG
>Unigene54535_BS
CAACATCCATACTCTCATGACCATAAGTACTACCATGAACCAAGCTCATCTTGGGGGAGATATACTCTTCCTCTCCTAGCATCTT
CTCCAACATCTCTGAGAATGTTAGTGAGCTGATTAGCAATTCCAAGGGCCAATGCAGCATTGTAAACGCTTTCTGTTGAGGCCC
TTGATTCTGGGCATATCCCCATCACCGGAACACTCATCAATCCAACAGTTCCAGCAACATAGTAGCAGTAAAGGTAAAGTTCAT
CAAAGTTCTGGTATCTTGACTTCCTCAAGTCTAATCTCATTCCTTCTACCATGTCTCTGAAGGGCTGAATGTCAACGGGGTACTT
GGTGACAGTATCAGATAGAGCAGCATCATACATATCGTAAGGTCGGCCTTCAAAGAGATCAGTCAGTCTTTTCTCCCATCTGTC
AAGAGCTTTGGGTGTAATGTATGAAGCATTAGGTCCATCCACTAGCTCATCTGTCCTTCTGCACCACACATAGATTGCCCAAAC
AGCTCGTCGCCGCTCCGGTGTCATGAGCAATGTGCCCAGGTAAAAA
>Unigene53723_BS
GACACACATTTGCCAACTACCACTGCTTTCAAAACTTTTGCCTTACCCTCCAATCTCCATAAGAGAAGCCACAACCTCACAGCT
CACATACTCACACCCTCACACTCAAAACCTAAAACCCAACAGATTAATTGTTCTTTTGATATGTCAGTTGTTCTTCTTTGGGTGG
TGAGCCCCAAACAGAATGCCAGCTCCCTGCTGGGTCTGATGCCCAGAATTTGCACCCCAAGGAGGTCTAAATTCTGCCCGAAG
CTGGGATTTTCAAGTAGGGTTTTGGCCTACTCGGGTGCAGTTGTAAACCCAGCAAGGTCTTCAGAAGAGAAGGTGTATGAAGT
GGTGCTGAAGCAGGCTGCGCTGGTGGAAGAACAGAGCACGGTAAAAAGGAAATCTTTGGATTTGGATGAACGGATTGTAACTG
AAGGTTTGGACAACTGGGATTTACTGGATAAGGCTTATGATCGGTGTGGTGAAGTCTGTGCTGAGTATGCCAAGACTTTTT

Supplementary Fig. S1. Unigene sequence of PSY in loquat RNA-Seq libraries.

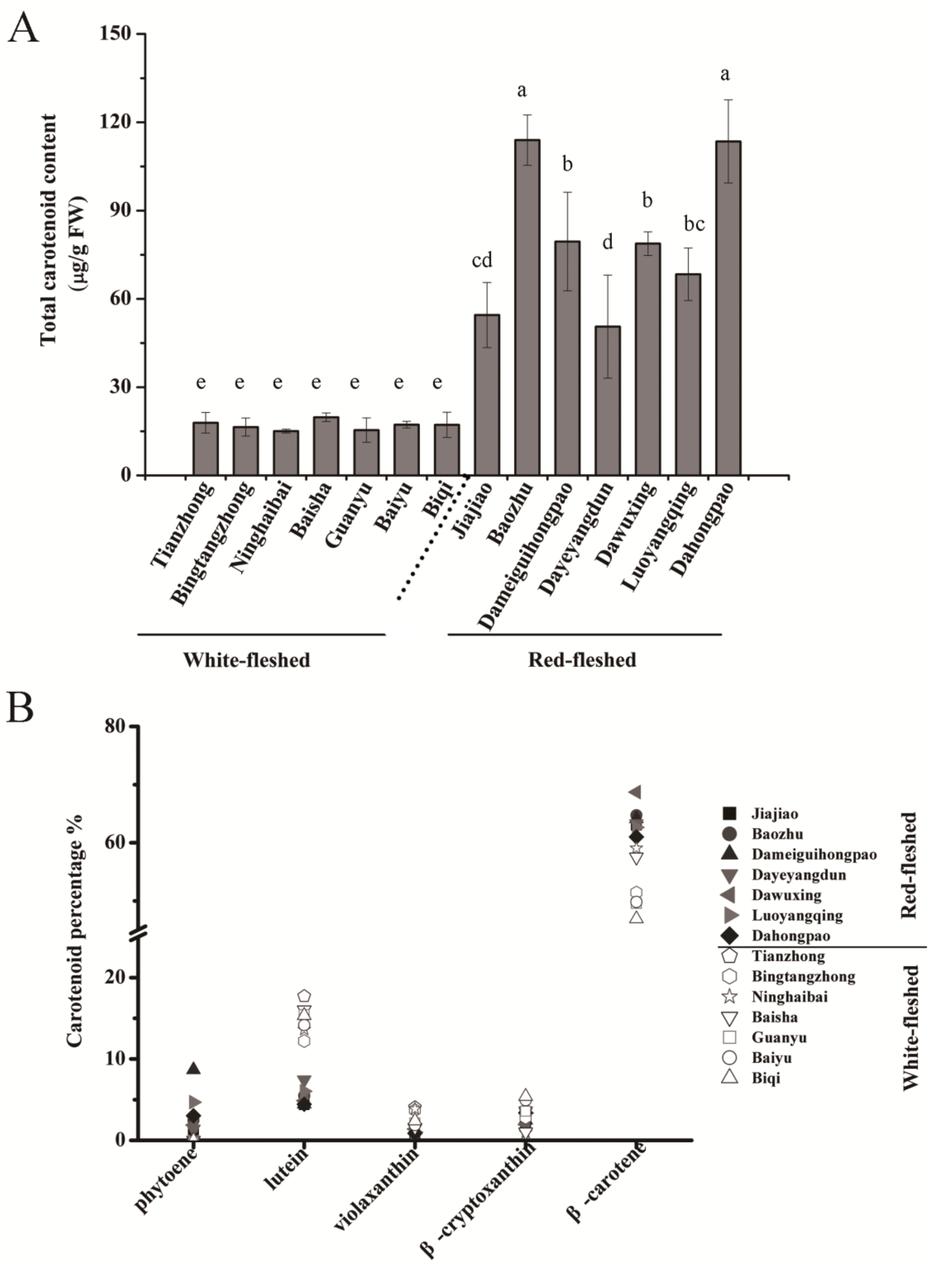

Supplementary Fig. S2. Carotenoid content and composition in the peel of red- and white-fleshed loquat cultivars. The percentage of the main carotenoids (phytoene, lutein, violaxanthin, β-cryptoxanthin and β-carotene) in the peel of red- and white-fleshed loquat cultivars was analyzed by HPLC.

EjPSY2A MSVVLLWVVSPKQNASSLLGLMPRICTPRRSKFCPKLGFSSRVLAYSGAVVNPARSSEEK
EjPSY2A^{d} MSVVLLWVVSPKQNASSLLGLMPRICTPRRSKFCPKLGFSSRVLAYSGAVVNPARSSEEK
****

EjPSY2A VYEVVLKQAALVEEQSTVKRKSLDLDERIVTEGLDNWDLLDKAYDRCGEVCAEYAKTFYL
EjPSY2A^{d} VYEVVLKQAALVEEQSTVKRKSLDLDERIVTEGLDNWDLLDKAYDRCGEVCAEYAKTFYL
****

EjPSY2A GTLLMTPERRRAVWAIYVWCRRTDELVDGPNASYITPKALDRWEKRLTDLFEGRPYDMYD
EjPSY2A^{d} GTLLMTPERRRAVWAIYVWCRRTDELVDGPNASYITPKALDRWEKRLTDLFEGRPYDMYD
****

EjPSY2A AALSDTVTKYPVDIQPFRDMVEGMRLDLRKSRYQNFDELYLYCYYVAGTVGLMSVPVMGI
EjPSY2A^{d} AALSDTVTKYPVDIQPFRDMVEGMRLDLRKSRYQNFDELYLYCYYVAGTVGLMSVPVMGI
****

EjPSY2A CPESRASTESVYNAALALGIANQLTNILRDVGEDARRGRVYLPQDELAQAGLSDDDIFRG
EjPSY2A^{d} CPESRASTESVYNAALALGIANQLTNILRDVGEDARRGRVYLPQDELGSW----------
***.

EjPSY2A KVTDKWQSFMKGQIQRARMFFDEAEKGVSELNSASRWPVWASLLLYRQILDVIEANGYDN
EjPSY2A^{d} --

EjPSY2A FTKRAYVGKAKKFVSLPVAYGRAIIGPSKLTKQLVPR-
EjPSY2A^{d} --------------------------------------

Supplementary Fig. S3. Comparison of the deduced amino acid sequences of EjPSY2A and EjPSY2A^{d}. Amino acid sequences were aligned using Clustal X

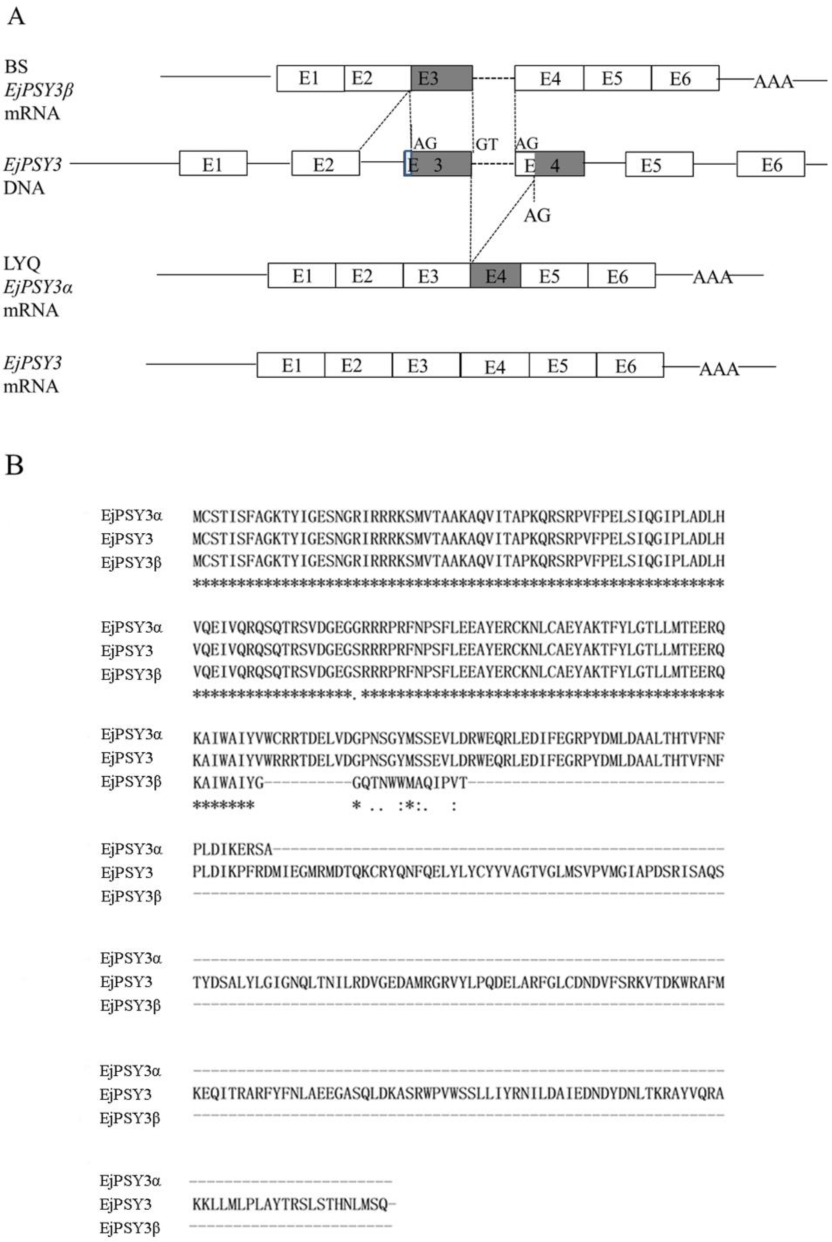

Supplementary Fig. S4. Analysis of the alternative splicing of EjPSY3α and EjPSY3β. (A) The differences in alternative splicing of *EjPSY3* in the LYQ and BS varieties. E, exon; AG, splicing acceptor site; GT: splicing donor site. In the LYQ variety 97 bases of the fourth exon were lost in *EjPSY3α*. In the BS variety, 10 bases of the third exon were lost and the former third intron was retained in *EjPSY3β*; Normal EjPSY3 mRNA was observed in the Jiajiao variety. The diagram is not to scale. (B) The amino acid sequence alignment of EjPSY3, EjPSY3α and EjPSY3β.

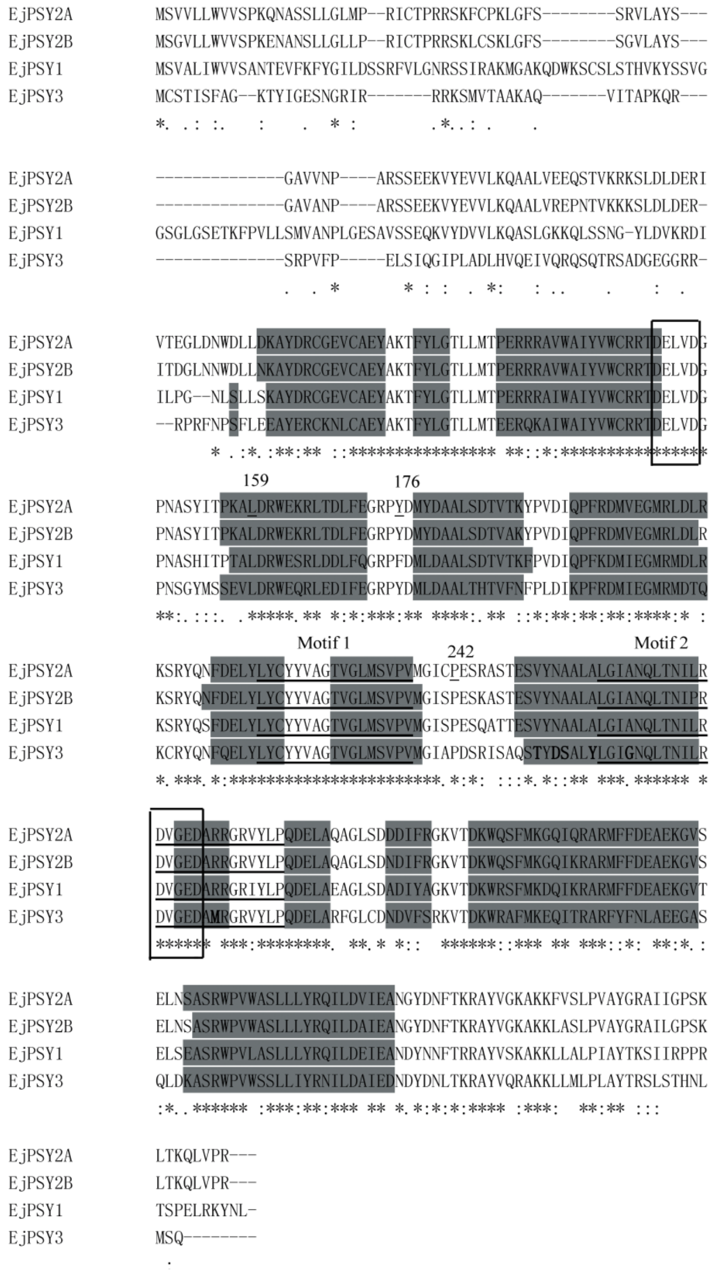

Supplementary Fig. S5. Alignment of EjPSYs amino acid sequences. Amino acids 159, 176 and 242 (underlined) have been shown to affect the activity or location of PSY in cassava (Welsh *et al*., 2010), tomato (Gady *et al*., 2011) and maize (Shumskaya *et al*., 2012); Underlining indicates PSY signature motifs 1 and 2 (conserved domain); Amino acids in bold in EjPSY3 sequence near or at motif 2 is different from other EjPSYs; Shading indicates α-helix, predicted by Swiss-model according to the structure of squalene synthase (PDB: 4HD1); Box indicates DXXXD putative active site. Amino acid numbers refer to EjPSY2A sequence.

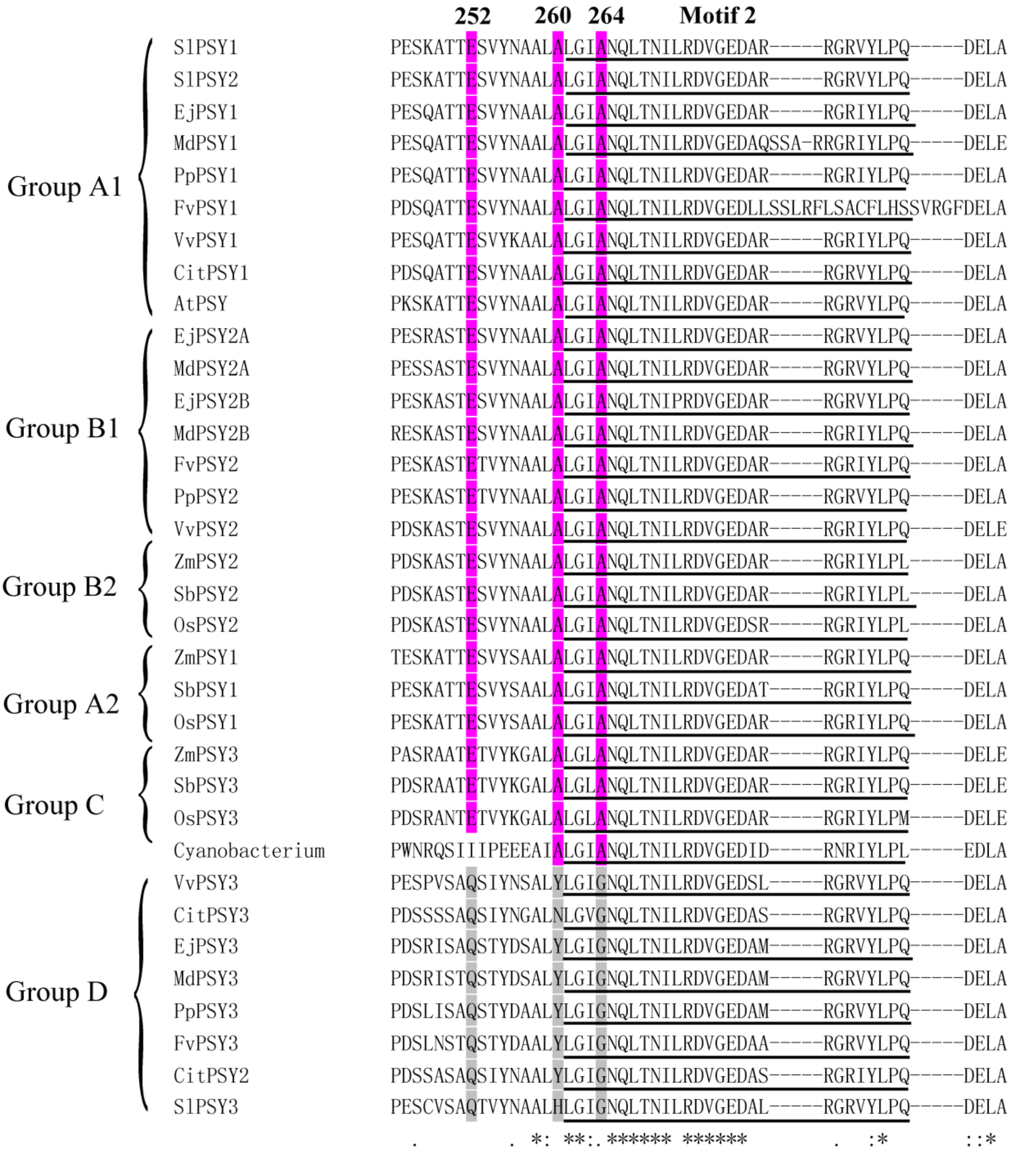

Supplementary Fig. S6. Alignment of PSY amino acid sequences. Alignment of PSY3s in dicot plants to all PSYs sequences available from the Phytozome; underlining indicates PSY signature motif 2 (conserved domain) (Shumskaya *et al.*, 2012); highlighting, amino acids 252, 260 and 264. Amino acid numbers refer to EjPSY3 sequence.

Supplementary Table S1. Primers for RACE PCR

Gene		5'RACE (5' to 3') primers	3'RACE (5' to 3') primers
EjPSY1	1st	TATCAGCGTCGGAGAGCCCTGCTTCT	GTTATGGGCATTTCACCTGAATCGCAA
	2nd	TTTGTTAGCTGATTCGCAATCCCTAAC	GCTGGGAAGGTCACAGACAAATGGAGG
EjPSY2A[d]	1st	TATCAGCGTCGGAGAGCCCTGCTTCT	CCCGTTGACATTCAGCCCTTCAGAGA
	2nd	TTTGTTAGCTGATTCGCAATCCCTAAC	ACCTTTACTGCTACTATGTTGCTGGA
EjPSY2B	1st	CTTATTCAGTAAATCCCAGTTGTTCA	TGAAGCAGGCTGCTCTAGTGAG
	2nd	AATCCCAGTTGTTCAAACCGTCAGTA	AGGCTGCTCTAGTGAGAGAACCGAA

Supplementary Table S2. Primer sequences for genome walking

Gene		Primers (5' to 3')
EjPSY1	1st	TTCTTTTTTACCGTGTTCGGTTCTCT
	2nd	AATATATATGCCTTCGCAAAGGGAT
EjPSY2A/*EjPSY2A*^{d}	1st	GCTGGCATTCTGTTTGGGGCTCACC
	2nd	AGTTTTGAAAGCAGTGGTAGTTGGC
EjPSY2B	1st	TTCCCGGGTAGAATAATATCCCGC
	2nd	ACTCAACTGCTTCTTACCCAAGGAT

Supplementary Table S3. Primers for genomic DNA PCR

Gene	Forward (5’ to 3’) primers	Reverse (5’ to 3’) primers
EjPSY1	TGATGAAACTGAATGAAATG	TTGAACTGGAATAAATAACACCCT
EjPSY2A/EjPSY2A[d]	ATTTGCCAACTACCACTGCTTTCA	TACACCACATAAGAAACAAGCA
EjPSY2B	ACCCAAAACCCCAAAGTCCCAGC	CCTCAGTTTGTCGCCCAACTAAC
EjPSY3	CTTCCAAATGTGTTCTACAATTTC	TGTTTTTATTATTGGGACATCAA

Supplementary Table S4. Primer sequences for PCR amplification of the *EjPSY2A*/*EjPSY2A*^{d} genomic fragments

Gene		Primers (5' to 3')
EjPSY2A/*EjPSY2A*^{d}	EjPSY2AUP1	TATGAACCATTGATTAGTCTAGC
	EjPSY2ADP1	GTTATTGTCACCGTAGTCGC

Supplementary Table S5. Primers for real-time PCR

Gene	Forward (5' to 3') primers	Reverse (5' to 3') primers
EjPSY1	GGGAGTGACAGAGTTGAGTGA	ATACGCAATAGGCAAGGCAAG
EjPSY2A	GGATATGCCCAGAATCAAG	CTTCCCGCGAAAGATGTCATC
EjPSY2A[d]	GGATATGCCCAGAATCAAG	TAAGTACTACCATGAACCAA
EjPSY2B	GGGAAAAGCAAAGAAGTTAGC	AGATTGAGACCTCAAAATAC
EjPSY3/*EjPSY3α*/*EjPSY3β*	CGGTTTGGGTTGTGCGAT	AAAGGCAGCATGAGAAG

Supplementary Table S6. Primers for PSY cDNAs subcloned as in-frame translational fusions

Gene	Forward (5' to 3') primers	Reverse (5' to 3') primers
EjPSY1	**GAATTC**GTTTTCAAGTTGAAAC	**GTCGAC**TCCAGCATTTTGTTG
EjPSY2A	CCG**GAATTC**ATGTCAGTTGTTCTTCT	**GTCGAC**TCATCTAGGCACCAACT
EjPSY2A[d]	CCG**GAATTC**ATGTCAGTTGTTCTTCT	**GTCGAC**CTACCATGAACCAAGCT
EjPSY2B	CCG**GAATTC**ATGTCAGGTGTTCTTCT	**GTCGAC**TCATCTAGGCACCAACT
EjPSY3/*EjPSY3α*/*EjPSY3β*	GAATT**GGATCC**TTCCAAATGTGTTCTAC	ATGAT**GTCGAC**ATAGCTCGAATACTCGC

Supplementary Table S7. Carotenoid content in peel and flesh tissues of white- and red-fleshed loquat cultivars

		Carotenoid content (μg g^{-1} FW)	phytoene	lutein	violaxanthin	β-cryptoxanthin	β-carotene	Total carotenoids
Peel	White-fleshed	Tianzhong	0	3.15±0.79	0.75±0.12	0.69±0.23	7.80±1.35	17.91 ±3.48
		Bingtangzhong	0	1.99±0.37	0.60±0.09	0.46±0.17	8.47±1.70	16.45 ±3.06
		Ninghaibai	0	2.04±0.14	0.58±0.04	0.14±0.02	8.88±0.28	15.05 ±0.66
		Baisha	0	3.20±0.67	0.32±0.12	0.21±0.05	11.35±0.75	19.77 ±1.48
		Guanyu	0	2.44±0.56	0.37±0.10	0.56±0.09	7.47±1.91	15.43 ±4.13
		Baiyu	0	2.50±0.10	0.35±0.17	0.92±0.09	8.52±0.52	17.26 ±1.13
		Biqi	0	2.72±0.37	0.59±0.14	0.90±0.09	7.91±1.95	17.19 ±4.28
	Red-fleshed	Jiajiao	0.41±0.09	2.31±0.19	0.26±0.08	1.18±0.28	33.89±5.06	54.51 ±11.07
		Baozhu	2.76±0.32	6.07±1.61	0.88±0.35	3.53±0.02	73.65±4.08	113.94 ±8.58
		Dameiguihongpao	6.78±0.91	3.38±0.08	1.09±0.29	1.89±0.03	51.08±8.26	79.50 ±16.77
		Dayeyangdun	0.66±0.12	3.84±1.52	0.80±0.44	1.79±0.51	31.75±5.39	50.56 ±17.48
		Dawuxing	1.50±0.15	3.80±0.28	1.09±0.01	1.54±0.09	53.98±0.19	78.75 ±3.99
		Luoyangqing	3.12±0.51	4.18±1.11	0.69±0.18	2.34±0.75	42.78±5.35	68.37 ±8.93
		Dahongpao	3.49±0.98	4.85±0.55	0.98±0.11	3.72±0.30	69.24±6.03	113.51 ±14.13
Flesh	White-fleshed	Tianzhong	0	0.05±0.01	0.07±0.00	0.13±0.04	0.06±0.02	0.56 ±0.10
		Bingtangzhong	0	0.05±0.02	0.06±0.03	0.16±0.07	0.07±0.03	0.67 ±0.27
		Ninghaibai	0	0.03±0.01	0.01±0.00	0.04±0.00	0.02±0.00	0.16 ±0.04
		Baisha	0	0.04±0.01	0.03±0.01	0.05±0.00	0.03±0.01	0.23 ±0.05
		Guanyu	0	0.02±0.01	0.02±0.01	0.08±0.03	0.05±0.02	0.33 ±0.12
		Baiyu	0	0.01±0.01	0.02±0.02	0.04±0.02	0.02±0.02	0.21 ±0.01
		Biqi	0	0.03±0.00	0.05±0.01	0.21±0.03	0.07±0.01	0.69 ±0.10
	Red-fleshed	Jiajiao	0.06±0.03	0.01±0.00	0.23±0.03	0.46±0.01	2.75±0.03	5.30 ±1.15
		Baozhu	0.34±0.15	0.05±0.02	0.80±0.29	2.83±0.94	3.69±0.38	12.43 ±2.69
		Dameiguihongpao	0.97±0.64	0.05±0.15	1.10±0.34	2.13±0.64	3.68±0.64	14.41 ±3.46
		Dayeyangdun	0.38±0.13	0.00±0.00	0.68±0.13	0.97±0.13	4.03±0.13	9.95 ±0.61
		Dawuxing	0.06±0.01	0.00±0.00	0.62±0.01	0.85±0.01	1.51±0.01	5.38 ±1.59
		Luoyangqing	0.75±0.11	0.10±0.02	0.94±0.21	2.34±0.16	2.66±0.50	11.54 ±2.29
		Dahongpao	0.39±0.08	0.05±0.01	0.73±0.01	2.35±0.35	2.91±0.65	11.96±1.30

± = Standard deviation (n=3)

Supplementary Table S8. Carotenoid content in various tissues of Luoyangqing (LYQ) and Baisha (BS)

Carotenoid content ($\mu g\ g^{-1}$ FW)	Peel of green fruit		Peel of ripe fruit		Flesh of green fruit		Flesh of ripe fruit		Young leaf		Mature leaf	
	LYQ	BS	LYQ	BS	LYQ	BS	LYQ	BS	LYQ	BS	LYQ	BS
neoxanthin	0.12±0.03	0.14±0.02	0.15±0.07	0.09±0.02	0.11±0.01	0.02±0.01	0.04±0.01	0.01±0.00	0.14±0.04	0.15±0.02	0.24±0.04	0.23±0.05
violaxanthin	0.52±0.12	0.40±0.15	2.68±0.55	0.68±0.28	0.14±0.05	0.03±0.01	0.50±0.09	0.03±0.01	4.17±1.23	3.48±0.98	21.60±3.25	17.88±2.88
luteoxanthin	0.42±0.13	0.29±0.04	0.53±0.15	0.56±0.29	0.03±0.01	0.05±0.02	0.35±0.10	0.02±0.00	0.99±0.32	0.91±0.17	13.28±2.26	10.10±1.97
9-*cis*-violaxanthin	0.03±0.01	0.04±0.01	0.69±0.18	0.32±0.12	0.07±0.02	0.03±0.01	0.94±0.41	0.03±0.01	1.11±0.31	0.86±0.28	7.55±1.99	5.43±0.74
lutein	9.58±2.51	5.75±1.16	4.18±1.11	3.20±0.67	0.37±0.09	0.16±0.00	0.10±0.06	0.04±0.01	8.03±1.96	6.41±1.23	57.38±6.57	48.87±5.54
phytoene	0.09±0.02	0.03±0.02	3.12±0.51	0.04±0.05	0.00±0.00	0.00±0.00	0.75±0.11	0.00±0.00	0.03±0.01	0.00±0.00	1.32±0.03	1.77±0.27
phytofluene	0.02±0.00	0.00±0.00	0.62±0.12	0.00±0.00	0.00±0.00	0.00±0.00	0.17±0.02	0.00±0.00	0.01±0.00	0.00±0.00	0.11±0.01	0.10±0.00
β-cryptoxanthin	0.00±0.00	0.00±0.00	2.34±0.75	0.21±0.05	0.02±0.00	0.00±0.00	2.34±0.16	0.05±0.00	0.00±0.00	0.00±0.00	0.00±0.00	0.00±0.00
ζ-carotene	0.00±0.00	0.18±0.01	0.63±0.16	0.00±0.00	0.00±0.00	0.00±0.00	0.29±0.11	0.00±0.00	0.00±0.00	0.00±0.00	0.00±0.00	0.00±0.00
β-carotene	3.25±0.93	1.76±0.22	42.78±5.35	11.35±0.75	0.52±0.12	0.14±0.03	2.66±0.50	0.03±0.01	2.84±0.33	2.02±0.99	32.69±4.89	25.81±3.38
unidentified	2.64±1.13	0.82±0.22	3.29±2.37	3.40±0.42	0.16±0.05	0.07±0.02	3.05±1.18	0.03±0.02	2.40±0.66	2.46±0.71	12.21±2.12	9.17±1.17
Total carotenoids	16.67±2.88	9.41±1.35	68.37±8.93	19.77±1.48	1.43±0.36	0.50±0.15	11.54±2.29	0.23±0.05	39.92±8.78	33.96±6.55	146.37±1.41	119.36±5.64

± = Standard deviation (n=3)
